# A System of Surgery

**Published:** 1847-12

**Authors:** 


### ARTICLE XIII.

*A System of Surgery*. By J. M. Chelius, Doctor in Medicine and Surgery, Public Professor of General and Opthalmic Surgery, etc., etc. Translated from the German, and accompanied with additional notes and observations, by John F. South, late Professor of Surgery to the Royal College of Surgeons, and one of the Surgeons of St. Thomas' Hospital. In three volumes, pp. 942, 640, 586. Philadelphia: Lea & Blanchard. 1847. (From the Publishers.)

The appearance of an elaborate treatise on Surgery, translated from the German, is an event of rare occurrence in medical literature, and deserves to be hailed with welcome by the profession. Even if the merits of the work was less than those of "Chelius' System," it would be a valuable addition to our libraries by making us acquainted with the principles and practice of a country where surgery has lately been cultivated with great success, and of which we hear directly so little. But this work, from the limited examination we have been able to give it, seems to be the most extensive and complete system of surgical practice in the English language, unless Cooper's Surgical Dictionary may be considered an exception, and in many respects, particularly in regard to recent improvements, this is less complete than that before us. The notes constitute indeed a large part of the work, and taken as a whole, it might be said to be almost as much English as German, and although they are in general judicious, they are not unfrequently in conflict with the text, a circumstance which, while it might be no disadvantage to the experienced surgeon, would be embarrassing to the younger student.

Truth to say, the plan so much adopted of late of adding notes to medical books, equal in volume to the original work, is liable to serious objections. It has doubtless arisen from the difficulty of embracing more than a very imperfect account of a subject within the limits proposed, and from a desire on the part of those acting as editors to supply these inevitable deficiences, and is to this extent, commendable. But when we see three corpulent tomes of 1000 pages each, and an atlas upon operations alone, and each succeeding treatise more voluminous than the preceding, we are driven to the conclusion that surgical science has become too extensive for text books, and can only be properly treated in a series of monographs,on each individual division of the subject. Inflammations, dislocations, fractures, &c., are subjects which might profitably occupy the attention of any individual member of the profession, and a treatise on which would be abundantly large for the student. While then, we look upon the publication of such works as the present as a great improvement on those mostly in use but a few years since, it appears that a still further advance is required in a separation of the different branches of the subject, and a consequent division of the labor.

It is but justice to say that occasions for animadversion on the practice of overloading books with notes more appropriate than the present, have often presented themselves, but the precise cause of the difficulty, and the remedy, were not then so obvious to our vision as at present, for as we have already stated, these volumes form perhaps the most complete system we possess, enriched with a copious bibliography and references to the authorities in all cases—these references when deficient in regard to American works, being completed by Dr. Norris. D. B.

